# Care of Children

**Published:** 1883-02

**Authors:** 


### CARE OF CHILDREN.

Every mother should know that very young children often suffer for the want of fresh, cold water. This they should have every two hours, and more frequently, if they become restless and fretful. Fretfulness is generally caused by great thirst. While making a voyage at sea some years ago, I was greatly disturbed by the incessant crying, or moaning, of a child, during the first few days of the journey. The child was about one year old and very delicate. I suggested to the mother that it wanted water. She said that it never, to her knowledge, tasted of water, as she thought it dangerous to give it to children so young. I assured her she need have no fear. I directed that it should have water every hour, a little at a time, until it became accustomed to it. This was given it, and during the rest of the voyage, of five or six days, I never saw a better or happier child. I believe the child's health was permanently impaired by the constant thirst that during its short life had been consuming it.

